# Primary care gatekeeping during the COVID-19 pandemic: a survey of 1234 Norwegian regular GPs

**DOI:** 10.3399/BJGPO.2023.0095

**Published:** 2024-03-06

**Authors:** Børge Lønnebakke Norberg, Tor Magne Johnsen, Eli Kristiansen, Frode Helgetun Krogh, Linn Okkenhaug Getz, Bjarne Austad

**Affiliations:** 1 Norwegian Centre for E-health Research (NSE) and General Practice Research Unit, Department of Public Health and Nursing, Norwegian University of Science and Technology (NTNU), Trondheim, Norway; 2 TillerTorget Medical Centre, Trondheim, Norway; 3 Midtbyen Medical Centre, Trondheim, Norway

**Keywords:** general practice, family medicine, primary health care, gatekeeping, COVID-19, pandemics

## Abstract

**Background:**

In the Nordic healthcare systems, GPs regulate access to secondary health services as gatekeepers. Limited knowledge exists about the gatekeeper role of GPs during public health crises seen from the perspective of GPs.

**Aim:**

To document GPs’ gatekeeper role and organisational changes during the initial COVID-19 lockdown in Norway.

**Design & setting:**

A cross-sectional online survey was addressed to all regular Norwegian GPs (*n* = 4858) during pandemic lockdown in spring 2020.

**Method:**

Each GP documented how patients with potential COVID-19 disease were triaged and handled during a full regular workday. The survey also covered workload, organisational changes, and views on advice given by the authorities.

**Results:**

A total of 1234 (25.4%) of Norway’s GPs participated. Together, they documented nearly 18 000 consultations, of which 65% were performed digitally (video, text, and telephone). Suspected COVID-19 symptoms were reported in 11% of the consultations. Nearly all these patients were managed in primary care, either in regular GP offices (55.7%) or GP-run municipal respiratory clinics (40.7%), while 3.7% (*n* = 73) were admitted to hospitals. The GPs proactively contacted an average of 0.8 at-risk patients per day. While 84% were satisfied with the information provided by the medical authorities, only 20% were able to reorganise their practice in accordance with national recommendations.

**Conclusion:**

During the early stage of the COVID-19 pandemic in Norway, the vast majority of patients with COVID-19-suspected symptoms were handled in primary care. This is likely to have protected secondary health services from potentially detrimental exposure to contagion and breakdown of capacity limits.

## How this fits in

GPs play an essential role as gatekeepers in protecting secondary health services in the Nordic healthcare system, but there is sparse research about this role during a public health crisis. This paper describes how GPs triaged and managed patients with suspected COVID-19 and handled other patients during the first societal lockdown in Norway in Spring 2020. The vast majority were managed in primary care, and only 3.7% of suspected cases were admitted to hospitals, indicative of well-functioning protection of hospitals. In 9.0% of non-COVID-related consultations, the GPs were concerned about delayed treatment for patients with potential severe diseases. The findings highlight the relevance of strong and flexible primary health care.

## Introduction

In Nordic healthcare systems, GPs are assigned an essential role as gatekeepers in regulating access to secondary health services. Research on the gatekeeper function has mostly examined the role of GPs under stable societal conditions, affirming a relationship between a strong primary healthcare system and reduced morbidity, mortality, and even increased life expectancy.^1,2^ The Norwegian regular GP scheme is briefly described in Box 1.

Box 1 **The Norwegian GP scheme**

The Norwegian healthcare system is based on the principles of universal access and continuity of care.^35^ Since 2001, all Norwegian citizens may sign up with a GP (and change, if desired), and 99% have chosen to do so, although 250 000 people are currently on a waiting list. The system is financed by taxation, together with income-related employee and employer contributions and out-of-pocket payments (co-payments). Private medical insurance is limited. While national healthcare policy is controlled centrally, responsibility for the provision of primary health care is decentralised. GPs act as coordinators of municipal services and gatekeepers to specialised care. On average, a GP has a list of approximately 1050 patients and often provides other medical services in the municipality 1 day a week. In addition, GPs generally take part in 24-hour emergency care services and many voluntarily participated in municipal-run respiratory clinics during pandemic lockdown.^36^

Earlier outbreaks of infectious diseases sparked interest in how the primary healthcare service is reorganised to optimise triage and avoid hospital overload during infectious health crises. It is recommended to deliver targeted preventive advice, inform patients about current public health guidelines, and reach out to vulnerable patients regarding the potential risk for contagion. Furthermore, it is essential to ensure that health care is provided to patients with other serious diseases as part of the general medical service during crises.^3–5^

The COVID-19 outbreak in 2020 widely challenged primary healthcare services^6,7^ and reignited the discussion about the role of primary healthcare services in protecting hospitals from overload.^8–11^ Areas with weak primary health care experienced hospital overload and increased mortality.^8,12^ In Italy, where early European cases of COVID-19 arose, a lack of trustful communication between the GP service and the authorities was reported.^13^ While the healthcare system was dealing with a vast number of patients with COVID-19, other groups of patients chose to stay home. In England, fewer patients consulted GPs with symptoms that could potentially indicate cancer, giving rise to concerns about delayed diagnosis.^14^ A recent Canadian study found that family physicians all over the country were not well incorporated into the COVID-19 pandemic response.^15^

In Norway, several societal preconditions influenced the course of the COVID-19 pandemic (Box 2). A first societal lockdown occurred between 12 March and 15 July 2020. Both the Norwegian authorities and the Norwegian Medical Association immediately established information channels to reach GPs. Many primary care practices stopped offering routine physical appointments. This was economically feasible, as the authorities introduced reimbursements for digital triage and consultations using telephone, video, and text-based e-consultations (that is, freely formulated questions and answers). Within 2 months, more than 80% of GP practices had implemented digital consultations, and six of 10 consultations were performed digitally during the study uptake.^16–18^ Most patients who attended physical consultations had either undergone digital triage or resided in geographical areas with a negligible risk of infection. A Norwegian study has documented that many GP offices lacked personal protective equipment to meet the COVID-19 pandemic so that the staff experienced fear of not being able to diagnose and treat patients safely.^19^

Box 2 **Contextual factors affecting the course of the COVID-19 pandemic in Norway when our study was conducted**

- An established welfare state and publicly organised health care: ensured rapid adaptation of healthcare reimbursement systems and implementation of digital care, paid sick-leave for registered workers in all sectors, support for financially threatened businesses, and so on.
- High public trust and social cohesion: as a nation, Norway is characterised by high trust in the authorities, social equalisation, and a tendency to follow expert advice.^37^
- Clear communication to the general public: from the start of the pandemic the Norwegian Ministry of Health established an authoritative and clear information strategy, providing advice on how to behave in the lockdown situation and tackle eventual symptoms.
- Societal lockdown: closure of schools, working from home if possible, regulations regarding physical distancing, social gatherings and travel, and so on.
- Establishment of effective communication channels to the healthcare sector: both the **Norwegian** Ministry of Health and the Norwegian Medical Association rapidly developed systems for communicating updates and recommendations regarding handling of the COVID-19 pandemic. These systems were in place but not fully developed when data collection for the present study took place.
- Demographic characteristics: in a European perspective, Norway is an affluent country with a relatively dispersed population of approximately. 5.4 million inhabitants, with 83% living in urban regions. The age distribution and proportion of immigrants is comparable with many European countries.^38^

In addition, GP-run respiratory clinics, also known as ‘fever clinics’, were rapidly established in most municipalities to offer safe clinical evaluations while minimising the spread of infection in general healthcare facilities. The respiratory clinics were mostly run by GPs pertaining to the regular GP scheme.^18^

Eventually, most Norwegian GP practices adapted to the COVID-19 pandemic in terms of infection control measures and organisational changes.^16^ During the whole period, consultations related to pandemic-related issues (COVID-suspected symptoms, concerns and infection control information regarding COVID-19) were free of charge for patients.^20^

The early lockdown period in 2020, when digital consultations were used for an unprecedented, broad range of clinical problems, reflects a unique transition period in the delivery of primary health care.^15^ Knowledge gathered during this period may have high future relevance for pandemics or other abrupt societal changes. To the best of our knowledge, our study is unique, as it builds on real-time data collected during lockdown.

The aim of the present study was to document the gatekeeper role of and organisational changes by GPs during the initial COVID-19 lockdown in Norway.

## Method

### Setting, study design, and data collection

A cross-sectional online survey was addressed to all Norwegian GPs (*n* = 4858) between 14 April and 3 May 2020 during the first national COVID-19 pandemic lockdown. The questionnaire contained 170 items. First, each responder answered generic questions covering workload and change in practice organisation. Workload was measured by counting the number of consultations (physical, video, telephone, and text-based freely formulated questions and answers). Organisational changes were measured through questions on GPs’ number of days in practice and conditions to reorganise working practices in accordance with the national recommendations. To investigate GPs’ situations during lockdown, we included questions regarding perceived information and recommendations from the health authorities, GPs’ access to personal protective equipment, and personal concerns regarding COVID-19-infection risk.

Then, the GPs were asked to document all clinical activities on one typical full workday at the GP office. To investigate the gatekeeper function, the number of patients with suspected COVID-19 symptoms was registered, together with information on whether the patient was handled locally by the GP, referred to a GP-run municipal respiratory clinic, or admitted to a hospital. Furthermore, the GPs reported whether they proactively contacted vulnerable patients owing to risk of a serious course of eventual COVID-19, the number of patients or next of kin with questions and concerns about COVID-19, and the number of patients who received tailored advice related to COVID-19 in case of symptoms, concerns, or other needs for infection control information. The GPs also registered whether they were concerned about possible delayed somatic diagnosis or treatment (that is, related to cancer) and about patients’ lockdown-associated psychosocial difficulties. We consulted the Checklist for Reporting Results of Internet E-Surveys to develop the survey and report its results.^21^ The survey was pilot tested by a panel of experienced GPs. It took approximately 90 minutes to complete the full survey. Two articles about GPs’ reported use of video consultations have been published from the same material.^17,22^

To reach responders, we collaborated with Norwegian Health Informatics (NHI), a web-based portal that hosts an online clinical decision support product (NEL), to which approximately 98% of all Norwegian GPs subscribe.^23^ An invitation was sent to all NEL subscribers including a unique email link to the survey, ensuring both the authenticity and anonymity of the responders. In addition, we used large social media groups of GPs to stimulate participation and invite GPs who did not receive a personal email. Several reminders were sent by email and social media. Corrected for this, the response rate is 26% of the GPs who de facto received the invitation.The survey was conducted through Netigate, an application for internet surveys.

### Statistics

The data were analysed using SPSS (version 26.0). We excluded answers with extreme outliers that were most likely wrong (for example, 141 consultations on a working day; *n* = 3). Questions eliciting GPs’ viewpoints were scored on a 5-point Likert scale and then combined into three categories. More participants (*n* = 1234) completed the generic questions, including workload estimation, than registration of the full working day (*n* = 910). Therefore, we calculated the total number of consultations by multiplying the average consultations per day (19.7 based on all 1234) with the number of responses to the triage questions (901–910). We performed a multivariable regression analysis to investigate the effect of GPs’ gatekeeping in relation to experience, recourses to adaptation of practices according to the authorities’ recommendations, and how information from the authorities was received.

## Results

In total, 1234 of the 4858 invited GPs (25.4%) partici pated and answered the generic questions. Of those invited, 910 (18.7%) completed registration of the working day, and we received answers from between 904 and 913 GPs to the questions concerning gatekeeping. The 1234 GPs reported an average of 19.7 (95% confidence interval [CI] = 19.6 to 19.9) consultations per day. Our material of 901–910 responses therefore covers approximately 18 000 consultations (17 767–17 945). The characteristics of the responders are presented in Table 1.

**Table 1.** Characteristics of participating GPs (*n* = 1234)

Background characteristic	Participants
**Sex**	***n* (%**)
Female	674 (55)
Male	550 (45)
**Years of experience as a GP**	
0–5 years	269 (22)
6–10 years	266 (22)
11–20 years	365 (30)
>20 years	333 (27)
**Citizens in the municipality of the practice**	***n* (%**)
<10 000	212 (17)
10 000–50 000	439 (36)
50 000–100 000	199 (16)
100 000–500 000	283 (23)
>500 000	100 (8)
**Employment**	
Self-employed	973 (79)
Self-employed with municipal support	99 (8)
Municipally employed	119 (10)
Other type	40 (3)
**Number of patients on GP’s list**	
<800	210 (17)
800–999	221 (18)
1000–1999	342 (28)
1200–1599	400 (33)
>1600	57 (5)

Total responders: Sex *n* = 1224, Years of experience as a GP, *n* = 1233, Citizens in the municipality of the practice *n* = 1233, Employment *n* = 1231, Number of patients on GP's list *n* = 1230.

### Triage and treatment of potentially infected patients

On their documented working day, 11.1% (*n* = 1997) of the consultations were with patients with suspected COVID-19 symptoms. In Table 2, we show how these patients were handled. Nearly all these patients were managed in primary care, either in regular GP offices (55.7%) or GP-run municipal respiratory clinics (40.7%), while 3.7% (73/1997) were admitted to hospital, corresponding to 0.4% (73/17904) of the total number of consultations.

**Table 2.** GPs’ handling of the 1997 registered patients with suspected COVID-19 symptoms

	Consultations, *n*Total = **1997**	Average occurrence per day^a^(95% CI)	Proportion of patients, %
Handled locally by the GP	1112	1.2 (1.1 to 1.4)	**55.7**
Referred to and handled by GP-run municipal respiratory clinics^b^	812	0.9 (0.8 to 1.0)	**40.7**
Admitted to hospital from the GP office	73	0.08 (0.06 to 0.10)	**3.7**

^a^Based on an average of 19.7 consultations per day. ^b^Municipal respiratory clinics in primary care were run by GPs at suitable locations.

We performed a multivariable regression analysis to study whether admission rates to hospitals were affected by the GP’s work experience (±5 years), financial and practical resources for adapting practice in accordance with the authorities' recommendations, and how information from the authorities was received (see Supplementary Information S1). We found no differences in referral rates, as these background variables explained only 0.3% of the variation in referral rates (R^2^ = 0.003), and neither was statistically significant.

### Medical help and preventive care for vulnerable patients

During lockdown, the GPs reported selected aspects regarding the composition of patients. In Table 3, we present COVID-related issues and concerns during a full-working day. In 9.0% (1613/17866) of the consultations, the GPs reported concerns about potentially delayed diagnoses of serious non-COVID-related diseases such as cancer. Twice as many people received ‘tailored advice’ concerning COVID-19 than the number who presented with suspected COVID symptoms (4.5 versus 2.2 per day), and the GPs proactively contacted on average one (0.8) at-risk patient per day. Concerns for lockdown-associated psychosocial difficulties were reported at 2.3 (95% CI = 2.1 to 2.4) a day.

**Table 3.** COVID-related issues and concerns during a typical, full-working day during the first societal lockdown in Norway

COVID-19-related topic	Patients, *n*	Average occurrence per day (95% CI)^a^	Proportion of all consultations, %(*n* = 17 886)
Patients with suspected COVID-19 symptoms	1997	2.2 (2.0 to 2.4)	11.2
Patients or next of kin with questions and worries about COVID-19	3330	3.7 (3.5 to 3.8)	18.6
GPs concerned about possible delayed diagnosis or treatment of serious disease other than COVID-19	1613	1.8 (1.6 to 1.9)	9.0
GPs who gave tailored advice concerning COVID-19	4076	4.5 (4.2 to 4.8)	22.8
GPs who proactively contacted patients at risk of a serious course of COVID-19	730	0.8 (0.7 to 0.9)	4.1
GPs concerned with patients’ lockdown-associated psychosocial difficulties	2048	2.3 (2.1 to 2.4)	11.4

^a^Based on an average of 19.7 consultations per day

### Organisational changes

During the pandemic lockdown, the GPs reported a 12% increase in time devoted to direct patient contact (in contrast to municipal and administrative tasks, and so on) to a total of 4.6 weekdays: 3.5 days (95% CI = 3.4 to 3.6) at the GP office and 1.1 days (95% CI = 1.0 to 1.2) working from home.

**Figure 1.**
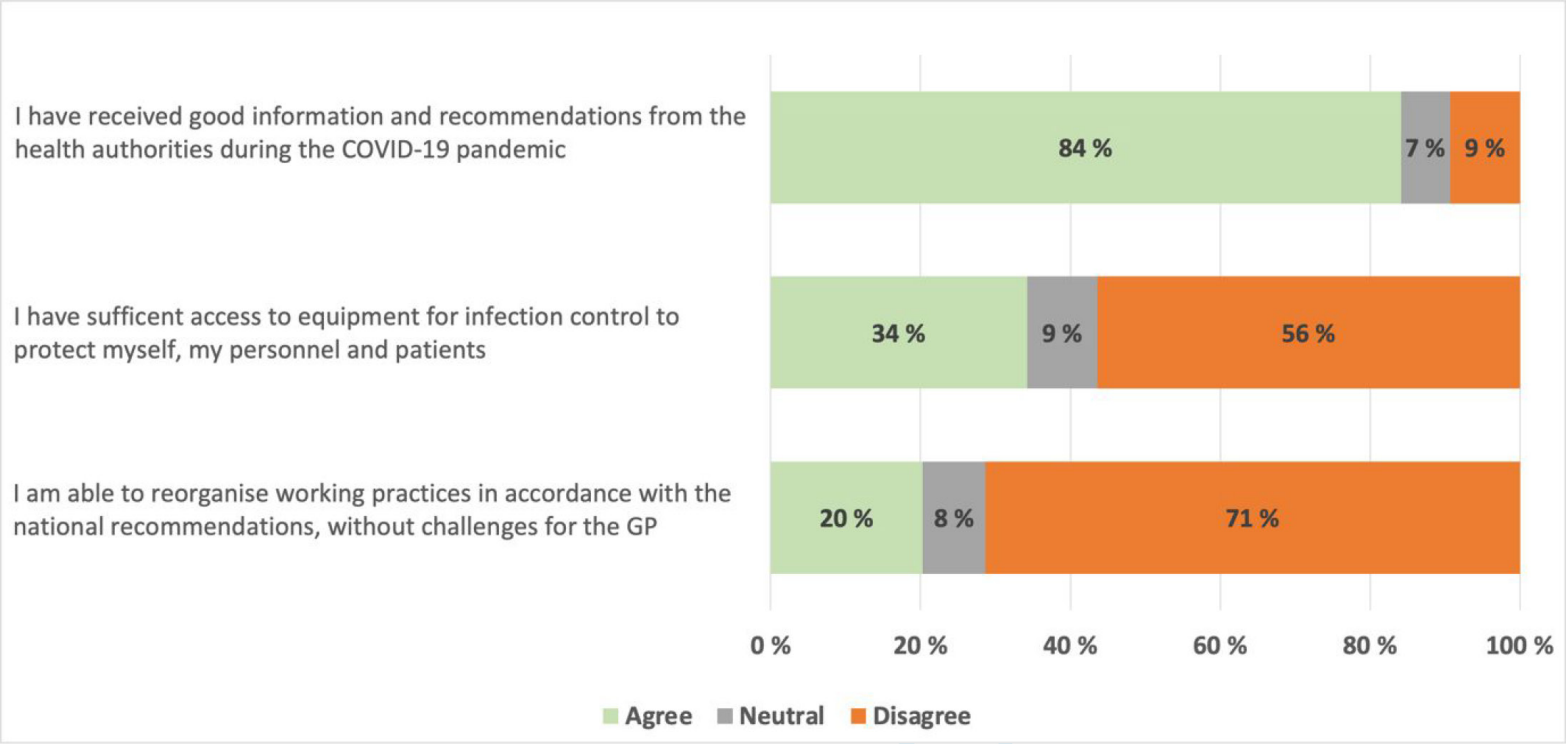
GPs’ viewpoints on pandemic-related information, personal protective equipment, and reorganisation of their practices

In Figure 1, we show that pandemic-related information provided by the Norwegian health authorities and the Norwegian Medical Association combined was regarded as satisfactory by 84% of the GPs. However, 56% disagreed that they had sufficient personal protective equipment, and only 20% were able to reorganise their practice in full accordance with the national recommendations at this early stage of the pandemic.

**Figure 2.**
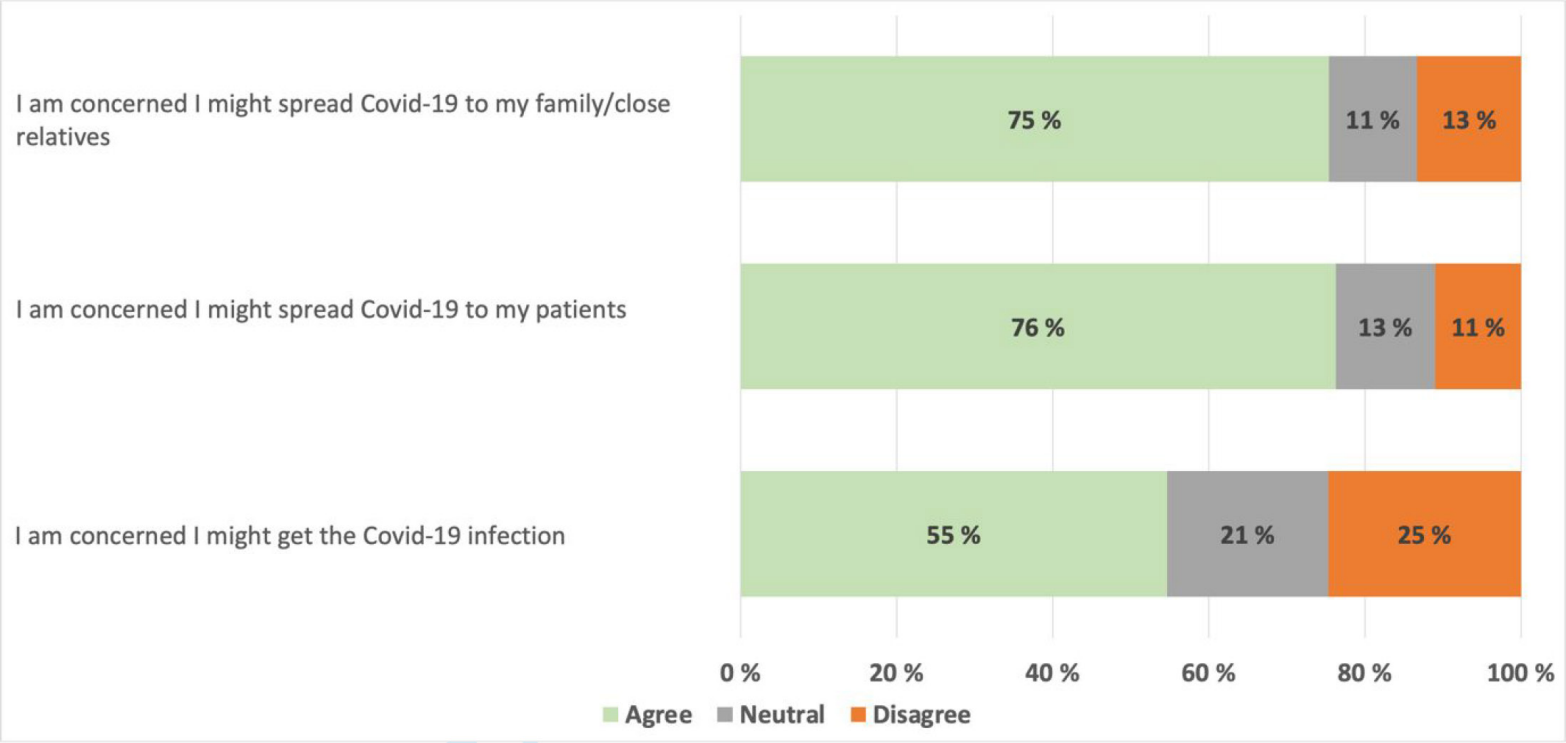
GPs’ concerns regarding infection risk

Figure 2, shows GPs’ personal concerns regarding COVID-19 infection in the early phase of the pandemic. While 76% were worried that they could spread the virus to their patients and 75% were concerned they would spread it to their family, 55% were worried about becoming infected themselves.

## Discussion

### Summary

Our study indicates that Norwegian GPs handled nearly all patients in the general population who sought medical attention owing to suspected COVID-19 symptoms during the pandemic. This reflects a clear gate-keeping function, suited to protect and diminish the pressure on hospitals. As responsible for defined patient lists, GPs proactively contacted vulnerable patients at particular risk of a serious course of COVID-19 while also striving to maintain health care for patients with severe non-COVID health problems. On a daily basis, GPs were concerned about delayed diagnosis and treatment owing to lockdown. We found a high degree of trust in the national authorities’ infection control advice, but only one in five GPs was able to reorganise their practices in accordance with national recommendations in the early stage of the pandemic.

### Strengths and limitations

Despite the fact that participation in our study took up to 90 minutes, we recruited one-quarter of all registered GPs in Norway during the exceptional period of pandemic societal lockdown. As previously discussed, our participants appeared relatively representative of regular GPs in Norway.^17^ Female and doctors aged <30 years were slightly overrepresented, as were GPs from Trøndelag County, where the research group is based.

Collaboration with NHI gave all subscribers an email with a unique link to our survey.^23^ This method made it slightly less accessible for the approximately 2% of GPs who do not subscribe to NHI. The use of Netigate prevented multiple answers from the same source**,** which is considered a strength. Alternative recruitment methods, such as through the Norwegian Medical Association, would likely encounter similar limitations. It can be assumed that their mailing lists do not encompass all Norwegian regular GPs.

National Norwegian data show that the total number of admissions per day with suspected COVID-19 peaked at 325 per day during early lockdown and then gradually decreased.^24^ Our findings of 73 admissions from one-fifth of Norway’s GPs of patients with suspected COVID-19 during early lockdown fits well with this, indicating reasonable representativeness of the entire population.

A few additional admissions directly from the respiratory clinics in these early weeks of lockdown, are not measured in this study, but can be considered negligible in number.

### Comparison with existing literature

Our study has added to the evolving literature about the role of primary care during the COVID-19 pandemic.^15,16,18,22,25^ By international comparison, Norway had very low and even subnormal mortality rates during the pandemic.^26^ This outcome has complex explanations,^26^ as outlined in Box 2 and a Norwegian report on the pandemic from 2022.^27^ It is beyond the scope of this paper to deliver exact estimates of the contribution of primary care. It is, however, relevant to note how the regular Norwegian GP scheme, based on continuity of care (Box 1), provided favourable premises for triage of COVID-suspect cases and preventive care for vulnerable patients in general, as recommended during infection outbreaks.^3,5^ Such emphasis was rapidly facilitated by Norwegian authorities through changes in the tariff system.^20^ Our data indicate that one in five patients who consulted their GP received tailored advice. This equalled twice the number of patients with suspected COVID-19 symptoms, indicative of efforts among GPs to provide preventive medicine to their list population. In Norway, patient selection was carried out using the GP’s electronic journals and various digital tools.^20^

While there was a general drop in hospital admissions, data from Statistics Norway show that the number of consultations in primary care was maintained during the pandemic.^27,28^ This validates our findings that the average GP conducted around 20 consultations on a typical workday, similar to pre-pandemic levels.^17,28^ The high level of digital consultations among the responders was similar to national numbers during this phase of the pandemic.^28^ The increase in reported clinical working hours despite an unchanged number of consultations is most likely explained by time-consuming information updates from the authorities and implementation of infection control measures.^29^

Our findings related to GPs’ worries about overlooking or undertreating potentially serious illnesses can be readily explained by restrictions during lockdown. Many of the digital consultations would, under normal circumstances, have been physical with possibilities for ordinary clinical examinations. The high focus on COVID-19 might have discouraged patients from seeking help for other types of problems.^14^

Trust in the medical authorities is central for a well-functioning healthcare system.^27^ Associations between patient trust in governmental information and high compliance have previously been found.^30,31^ Our finding that Norwegian GPs generally trusted information from the medical authorities and their medical association can be seen to reflect a well-functioning system for crisis-related communication in the healthcare system.

Our findings of a lack of personal protective equipment and difficulties reorganising GP practices in the early phase of the pandemic have also been shown by others.^16,18^ Lack of equipment might explain why so many GPs were concerned that they might get infected and spread COVID-19 to their patients and families. With sufficient equipment in the early phase of the pandemic, GPs could have handled more patients in their own offices, reducing the need for GP-run respiratory clinics.

Previous studies have indicated that experienced GPs tend to admit fewer patients to hospitals than inexperienced colleagues.^32,33^ Our findings, however, indicate that for COVID-related admissions during the pandemic, this might not have been the case. One explanation could be that COVID-19 was a previously unknown disease and admissions were to a high extent guided by algorithmic recommendations with limited room for clinical deliberations.^34^

### Implications for research and practice

Our study has highlighted the value of strong primary health care during public health crises. It has documented how regular GPs in Norway, based on defined patient list responsibility, could work effectively as gatekeepers to secondary care during a pandemic lockdown, while also remaining attentive to other patients’ important but less acute needs. Our study has further emphasised the need for useful information, trust in advice from the authorities, enough protective equipment, and resources to reorganise local GP practices. The findings have relevance for future health service planning with respect to public health crises.

## Data

The dataset generated and/or analysed during the current study is not publicly available but can be made available from the corresponding author on reasonable request.
